# Defining and detecting malaria epidemics in south-east Iran

**DOI:** 10.1186/1475-2875-11-81

**Published:** 2012-03-23

**Authors:** William R McKelvie, Ali Akbar Haghdoost, Ahmad Raeisi

**Affiliations:** 1 Diocese of Hyderabad, Ashraf Goth, Rattanabad, PO Box 21, Mirpur Khas 69000, Sindh, Pakistan; 2 Research center for modeling in health, Kerman University of Medical Sciences, Jomhoori Islami Blvd, Kerman, Iran; 3 School of Public Health, Tehran University of Medical Sciences, Tehran, Iran; 4 National Malaria Control Programme, Health Ministry of Iran, Hafez-Jomhoori, Tehran, Iran

**Keywords:** Malaria, Epidemics, Iran, Sensitivity, Specificity, Surveillance

## Abstract

**Background:**

A lack of consensus on how to define malaria epidemics has impeded the evaluation of early detection systems. This study aimed to develop local definitions of malaria epidemics in a known malarious area of Iran, and to use that definition to evaluate the validity of several epidemic alert thresholds.

**Methods:**

Epidemic definition variables generated from surveillance data were plotted against weekly malaria counts to assess which most accurately labelled aberrations. Various alert thresholds were then generated from weekly counts or log counts. Finally, the best epidemic definition was used to calculate and compare sensitivities, specificities, detection delays, and areas under ROC curves of the alert thresholds.

**Results:**

The best epidemic definition used a minimum duration of four weeks and week-specific and overall smoothed geometric means plus 1.0 standard deviation. It defined 13 epidemics. A modified C-SUM alert of untransformed weekly counts using a threshold of mean + 0.25 SD had the highest combined sensitivity and specificity. Untransformed C-SUM alerts also had the highest area under the ROC curve.

**Conclusions:**

Defining local malaria epidemics using objective criteria facilitated the evaluation of alert thresholds. This approach needs further study to refine epidemic definitions and prospectively evaluate epidemic alerts.

## Background

Malaria epidemics cause significant morbidity and often mortality where they occur [1-4]. To predict malaria epidemics, several methods with varying lead times and sensitivities have been proposed. For example, Malaria Early Warning Systems (MEWS) predict epidemics based on weather forecasts. They provide a longer lead time, but poor sensitivity and specificity. In contrast, Early Detection Systems (EDS's) raise an alert shortly after the onset of an epidemic, providing little or no lead time, but a more specific warning of an epidemic [1,5]. An alert is raised when a weekly case count exceeds the corresponding weekly threshold. Malaria Control Programmes (MCP's) then investigate or immediately implement epidemic control measures. Several different methods for calculating alert thresholds have been proposed [2-6]. These are ideally based on five years of historical surveillance data [7,8].

Before widely implementing any EDS, one needs to evaluate the validity of its alert thresholds [7]. Alert threshold validity, at least for other disease surveillance systems, is normally assessed by measuring sensitivity and specificity [9]. Sensitivity is the percentage of "true epidemic periods" which correctly raised an alert. Specificity is the percentage of non-epidemic periods which correctly did not to raise an alert. Lower thresholds increase sensitivity (so fewer epidemics are missed), but decrease specificity (so more false alarms are raised). Higher thresholds decrease sensitivity, but increase specificity. Finding the optimal alert threshold involves striking a balance between sensitivity and specificity.

To calculate the sensitivity and specificity of any EDS threshold, a gold standard definition of what constitutes a malaria epidemic is needed. Unfortunately, there is no universal consensus on how to define malaria epidemics [5,10]. Such a definition would have to distinguish true epidemics from seasonal variations and from random transient aberrations.

Only two studies have used standard sensitivities and specificities to evaluate EDSs, but these had important flaws in methodology [11] or data collection [12]. Other evaluations used non-standard measures of validity [10] or anecdotal reports [13]. Without a good way to evaluate EDS threshold validity, implementing an EDS in any epidemic-prone area will remain problematic.

Malaria has been eliminated from most of Iran so that now only 6% of the population lives in endemic areas [14]. Unfortunately there has been little decrease since 2002 [15]. Malaria persists in three of Iran's thirty provinces, all clustered in the south-east [14]. About 90% of Iran's malaria cases are detected in these provinces, (A. Raeisi, personal correspondence) and epidemics have been reported there [7,14]. The MCP needs to control malaria epidemics in these three provinces if it is to meet its goal of elimination. It already has an efficient case monitoring and reporting system, but lacks a formal threshold-based EDS [7].

This study developed a local definition of what constitutes a malaria epidemic in Chabahar District, south-east Iran by comparing how well several proposed definitions correlated with observed aberrations in transmission. Then alert thresholds were developed for the early detection of local epidemics. Finally, the sensitivity, specificity, and timeliness of those alert thresholds were evaluated using the local definition.

## Methods

### Study setting

This study analysed surveillance data from Chabahar, one of the endemic districts of Iran, collected between 21 March 2003 and 19 March 2008. Chabahar District is located in south-east Iran. It has a population of 204,587. On average, the health system detects about 2,000 malaria cases there annually.

Iran's malaria control and surveillance system is implemented mainly through the Primary Health Care (PHC) system so data collection covers the entire district. The PHC also dispenses all malaria treatment free of charge to both nationals and expatriates, even that prescribed by private doctors. All suspected cases are confirmed through quality assured smear microscopy and each of the district's 15 rural health centres reports any positive results to the District Health Centre, usually the same day [7,14,16].

### Data management

To address of the main objective of this study, records of symptomatic cases from all 15 regional malaria labs of Chabahar District were used. Because of their proximity, data from five labs in two areas were pooled by area, making 12 reporting centres. Names and addresses were stripped to preserve confidentiality and the spreadsheet was translated into English. The data were then exported into Stata version 8.2 for Windows [17]. Gregorian dates were generated and all variables appropriately cleaned and labelled, leaving the original dataset intact.

Weekly case counts were aggregated by health centre, species and transmission type. The analysis was limited to indigenous malaria cases, because they are a better reflection of local malaria transmission [18]. For each health centre, week (*t*) and year (*y*), weekly indigenous case counts (*w_t, y_*), were incremented by one and logarithms taken to give the log counts (*x_t, y_*). Finally, three-week moving averages of log counts were calculated (z_t, y_) to provide a time series of smoothed log counts. These baseline variables were used to help define epidemic and alert thresholds.

### Defining epidemics

Several epidemic variables were plotted against a times series of weekly case counts to compare how well they correlated with observed aberrations in transmission. The details of how they were calculated are described elsewhere [18]. Most of these consisted of both weekly and annual thresholds, and a minimum duration. The two definitions which on visual inspection appeared to best identify epidemics were selected for use in assessing validity of alert thresholds and for determining the effect of changing definitions on measures of validity.

### Defining epidemic detection (alert) thresholds

**Figure 1:**
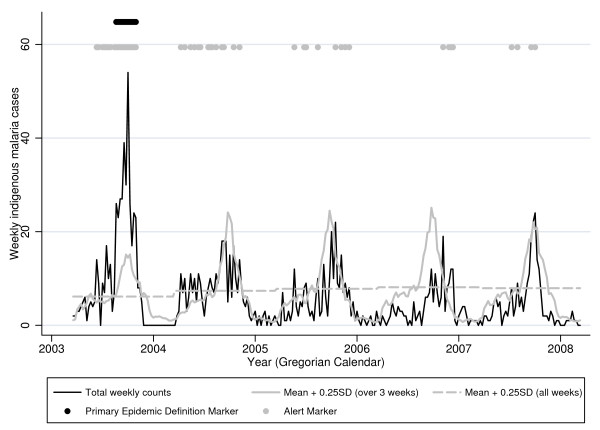
**Weekly indigenous malaria counts in Chabahar City (21 Mar 2003-19 Mar 2008)**. When weekly case counts (black line) exceed both the mean of baseline counts in the surrounding 3 weeks plus 0.25 standard deviations (solid grey line) and also exceed the mean over all weeks in the baseline plus 0.25 standard deviations (dashed grey line), an alert is triggered (grey dots at top of graph). The epidemic weeks according to the primary definition (weekly smoothed log counts exceeded both weekly and annual means plus 1 standard deviation for at least 4 weeks) are marked with black dots at the top of the graph.

Six different alert types were tested, each consisting of both a week-specific and an annual threshold. Positive alerts required that weekly counts exceeded both weekly and annual thresholds. For all six alert types, annual thresholds were calculated using all weekly counts in the baseline. Weekly thresholds were calculated in one of two ways: 1) for "one week span" alerts, weekly thresholds were calculated from baseline counts corresponding to the current week alone; 2) for "three-week span" alerts, weekly thresholds were calculated from baseline counts corresponding to the previous, current, and following weeks. Figure 1 shows how this worked at one centre for one alert type using a three-week span.

Annual and weekly alert thresholds were calculated from n^th ^percentiles (where n = 50, 60, 65, 70, 75, 80, or 85), from means plus *k *X standard deviation (*SD*), or from geometric means plus *k *X *SD *(where *k *= 0.25, 0.5, 1.0, 1.5, 2.0, 2.5, 3.0). For each of these three methods, weekly thresholds were generated from baseline data using either one-week or three-week spans as described above, giving six alert types in all. Alerts calculated from weekly means using one-week spans of baseline data were called modified Cullen alerts [6]. Alerts calculated from weekly means using three-week spans were called modified C-SUM (cumulative sum) alerts [2-5].

### Baseline data

To calculate means and standard deviations for both epidemic definitions and alert thresholds, the entire dataset excluding the year being tested was used as the baseline [10]. This allowed separation of test and baseline data, and inclusion of all epidemics periods. However, this approach tends to lower thresholds in epidemic years and increase thresholds in non-epidemic years, possibly inflating sensitivity and specificity. To assess this possible bias, calculations were repeated using the entire dataset without exclusion as the baseline [19].

### Statistical methods

For each alert type and threshold level, sensitivity was calculated as the percentage of epidemic weeks (according to the definition) with a positive alert and specificity as the percentage of non-epidemic weeks with a negative alert. The week of each epidemic when alerts first became positive was also determined.

Calculations were made initially using the primary epidemic definition noted above (counts exceed weekly and annual smoothed geometric mean plus 1 SD for at least four weeks) and baseline data which excluded the current year. Calculations were repeated using a three-week minimum epidemic duration and with baseline data which included the current year. To assess the effect of changing definitions or baseline data on the relative performance of alert types, areas under the curve (AUCs) of receiver operating characteristic (ROC) plots were calculated and compared.

## Results

### Total case counts, temporal trends, and seasonal variations

Between 21 March 2003 and 19 March 2008, 10,738 cases of malaria were reported in Chabahar District, of which 8,055 (75.0%) were *Plasmodium vivax*. Total reported cases in the study period varied between centres, ranging from 149 (from Kambelsoleiman) to 2,446 (from Chabahar City). Most reported malaria cases were indigenous (62.9%), of whom 67.3% were adults. Females comprised only 29.8% of total cases.

**Figure 2:**
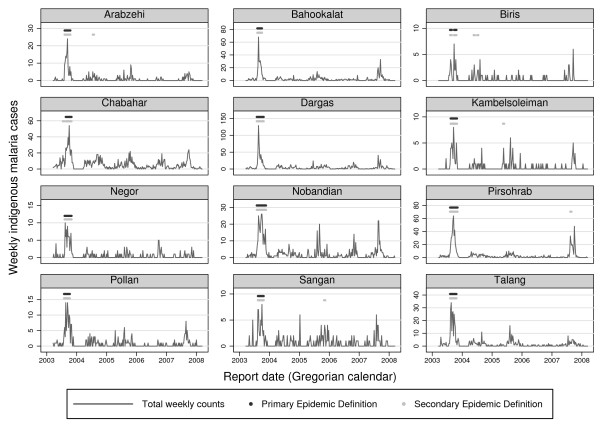
**Weekly indigenous malaria counts with epidemics marked in all centres of District Chabahar, (21 Mar 2003-19 Mar 2008).** For the primary epidemic definition, weekly case counts (black line) had to exceed both weekly and annual smoothed geometric means plus 1 standard deviation for at least 4 weeks (flagged by black dots at top of graphs). For the secondary epidemic definition used the same thresholds and a 3-week minimum duration (flagged by grey dots at top of graphs).

Marked inter-annual variations in transmission were observed. The highest number of cases occurred in the first year of the five-year study period: 4,007 (37.3%), over twice that of any other year in the period (see Figure 2). Annual cases then remained at a stable lower level for the remaining four years, showing no temporal trend (*p *= 0.77).

Total case counts varied considerably from week to week. Of the 3,120 weekly reports, 2,076 (66.5%) reported less than three cases and in 1,097 weeks (35.2%), no cases were reported at all. Both *Plasmodium vivax *and *Plasmodium falciparum *counts were much higher in August to October than in other months. Inter-annual variations were also highest at that time.

### Defining epidemics

The best definition of an epidemic was a period of at least four consecutive weeks in which smoothed weekly log counts (*z_t, y_*) all exceeded both the annual mean plus one standard deviation and *z_t, y _*also exceeded respective weekly mean plus one standard deviation. It flagged 13 epidemic periods lasting a total of 143 weeks. All occurred from August to October of the first year of the study period (see Figure 2). This was the primary definition used in the subsequent analysis.

The second best definition was like the primary definition except it used a 3-week minimum duration. This alternative definition flagged 20 epidemics lasting 164 weeks, but at least six of these flagged epidemics were spurious (see Figure 2). Other definitions that we examined performed much more poorly [18].

### Effects of changing baseline data on epidemic definitions and alert thresholds

Using a five-year baseline, the primary definition flagged the same 13 epidemic periods as when using the four-year baseline but only 128 epidemic weeks, 15 weeks less than with a four-year baseline. With the alternative definition, the number of epidemic periods decreased from 20 to 18 and epidemic weeks decreased from 164 to 143 using the larger baseline dataset.

Using a four year baseline, weekly alert thresholds in the first year (an epidemic year) were, as expected, lower than in other years, especially during seasonal peaks (see Figure 1). These differences reached statistical significance for both the primary definition (z = 3.13, *p *= 0.0017), and the alternative definition. (z = 3.80, *p *= 0.0001).

Using a five-year baseline epidemic thresholds were, of course, the same from year to year.

### Performance of alert thresholds

Using the primary epidemic definition (weekly counts > geometric means + ISD for ≥ 4 weeks) as the gold standard and a four-year baseline, all six types of thresholds were able to predict epidemics within two weeks at thresholds producing at least 90% specificity. The modified C-SUM alert using raw counts and a threshold of 0.25 standard deviations above mean gave the highest sum of sensitivity and specificity (see Table 1).

**Table 1:** Sensitivities, specificities, and detection times of various alert thresholds in Chabahar District, south-east Iran (21 Mar 2003-19 Mar 2008)

			Earliest week epidemic detected (n = 13)			
**Thresholds**	**Sensitivity** **(percent)**	**Specificity** **(percent)**	**1st**	**2nd**	**3rd**	**≥ 4th**

**Percentiles (1 week span)**						

> 60%	93.7	81.5	12	1	0	0

> 70%	93.7	85.5	12	1	0	0

> 75%	87.4	91.2	10	3	0	0

> 80%	74.8	94.3	5	6	1	1

**Percentiles (3 week span)**						

> 60%	96.5	81.7	12	1	0	0

> 70%	95.1	87.5	12	1	0	0

> 75%	94.4	90.5	12	1	0	0

> 80%	90.9	93.1	11	2	0	0

> 85%	78.3	95.6	6	6	1	0

**Mean count + SD's (1 week span)**						

> mean + 0.25SD	97.2	89.4	12	1	0	0

> mean + 0.50SD	93.7	93.4	10	3	0	0

> mean + 1.00SD	81.8	97.2	6	6	1	0

> mean + 1.50SD	69.9	98.2	4	6	2	1

**Mean count + SD's (3 week span)**						

> mean + 0.25SD*	**99.3**	**90.0**	**12**	**1**	**0**	**0**

> mean + 0.50SD	93.7	93.9	10	3	0	0

> mean + 1.00SD	83.9	97.3	7	6	0	0

> mean + 1.50SD	72.7	98.4	5	6	1	1

**Mean log(count + 1) + SD's (1 week span)**						

> mean + 0.25SD	98.6	78.2	12	1	0	0

> mean + 0.50SD	96.5	84.5	12	1	0	0

> mean + 1.00SD	84.6	93.0	9	3	1	0

> mean + 1.50SD	70.6	97.5	4	6	2	1

**Mean log(count + 1) + SD's (1 week span)**						

> mean + 0.25SD	100.0	77.1	13	0	0	0

> mean + 0.50SD	100.0	83.5	13	0	0	0

> mean + 1.00SD	93.0	92.9	10	3	0	0

> mean + 1.50SD	74.1	97.7	5	6	1	1

C-SUM alerts with raw counts also had the greatest area under the curve (AUC) of its ROC plot compared to other alert types (AUC = 0.9845, *χ*^2 ^= 67.95, *p *< 0.0001). The AUC of raw count C-SUM alerts decreased only slightly when calculated against a three-week minimum epidemic definition and still exceeded AUCs of other alert types: 0.9840 (*χ*^2 ^on 5 df = 63.25, *p *< 0.0001).

Using all five years of baseline data, the C-SUM AUC still exceeded those of thresholds based on percentiles or log counts. Surprisingly though, the AUC for raw count Cullen thresholds actually increased and exceeded that of C-SUM thresholds (see Table 2).

**Table 2:** Areas under the curve (AUCs) of ROC curves for various epidemic definitions and alert thresholds, Chabahar District, 21 Mar 2003-19 Mar 2008

Areas Under ROC Curves				
**Years of Baseline data**	**4 years (excludes test year)**		**5 years (includes test year)**	

**Minimum Epidemic Duration (Definition)**	**4 weeks (Primary)**	**3 weeks (Alternative)**	**4 weeks (Primary)**	**3 weeks (Alternative)**

**Threshold Type (span)**				

Percentiles (1 week)	0.9481	0.9492	0.9324	0.9321

Percentiles (3 week)	0.9624	0.9634	0.9469	0.9480

Mean+SDs (1 week) (Cullen alerts of raw counts)	0.9724	0.9702	**0.9743**	**0.9718**

Mean+SDs (3 week) (C-SUM alerts of raw counts)	**0.9845**	**0.9840**	0.9634	0.9659

Geometric mean+SDs (1 week) (Cullen alerts of log counts)	0.9647	0.9655	0.9607	0.9629

Geometric mean+SDs (3 week) (C-SUM alerts of log counts)	0.9768	0.9778	0.9510	0.9541

χ^2 ^for homogeneity (on 5 df's)	67.95	63.25	52.84	58.62

*P*-value	< 0.0001	< 0.0001	< 0.0001	< 0.0001

## Discussion

### Summary of findings

The primary epidemic definition (weekly smoothed log counts > weekly and annual means + 1SD, four-week minimum duration) was the most sensitive and specific, identifying 13 epidemics, though it may have missed a possible epidemic in the final year in Pirsohrab. It also remained robust to changing the baseline data.

Among alert types, raw count C-SUM alerts performed best. (These are thresholds derived from means and standard deviations of raw weekly counts from 3-week spans in the baseline data). Using a threshold of mean plus 0.25 standard deviations, C-SUM alerts detected 12 of 13 epidemics within one week and all within two weeks. This alert threshold had the highest combined sensitivity and specificity, 99.3 and 90.0 respectively. With changes in the baseline data, it still performed better than all other alert types except the Cullen type.

### Epidemic definitions

Distinguishing between malaria epidemics and normal seasonal or inter-annual variations can be challenging. Some have even eschewed defining malaria epidemics at all because no universally agreed criteria exist for declaring them [10]

Though no *universally *agreed definition exists, this study's *local *definition of malaria epidemics was based on several agreed principles. First, Chabahar District lies in an area prone to malaria epidemics. Applying malaria epidemic definitions to areas where they probably had not occurred had impaired earlier efforts to define epidemics [12].

This study also distinguished normal from excess transmission [8] by adapting EDS thresholds described by Abeku et al. [20] and distinguished epidemics from transient increases [8] by retrospectively setting a minimum duration [19]. A four-week minimum duration was chosen because four-week epidemics would peak at two weeks, the Abuja declaration's target for detecting epidemics [21]. This assumes that shorter epidemics would peak and begin to resolve earlier. To test this assumption, a definition with three-week minimum period was also assessed and found to be less specific, as expected. Other epidemic definitions examined were insensitive, non-specific, or much less robust to changes in the baseline dataset [18].

Finally, epidemics in this study were defined according to the epidemiology of malaria and available resources in Chabahar District. Hence, both *Plasmodium vivax *and *Plasmodium falciparum *were included rather than using only *Plasmodium falciparum *cases as was done in Ethiopia [19]. Also, only indigenous cases were included because there was no local evidence that increases in imported cases affected indigenous transmission, though imported cases may have precipitated epidemics elsewhere [22,23]. The lack of temporal trend and the presence of epidemic control measures in District Chabahar also influenced the definition.

All recognize that EDSs must be tailored to local epidemiological and social circumstances. Local circumstances should also dictate how one defines malaria epidemics. The lack of universally agreed criteria should not prevent defining malaria epidemics locally. Using specific criteria does not obviate the need to exercise judgement. The authors had to use judgement when assessing whether peaks in reported malaria cases were actual epidemics and whether epidemic definition variables correctly labelled them. However, objective criteria can inform judgement.

Teklehaimanot et al. [10] argued that one can avoid the problem of defining epidemics by comparing the validity of EDS thresholds using an alternative to sensitivity and specificity. There are three problems with this position. First, the agreed indicators of successful EDSs, detection and control of epidemics within two weeks [21] depend upon defining epidemics. Second, sensitivity and specificity are the standard measures of validity for alert thresholds of many diseases [9]. Third, the proposed alternative, "potentially preventable cases" (PPC), depends not only on the utility of the alert thresholds, but also on the lack of past epidemic control. As epidemics are contained, PPC will decline. Thus, PPCs would be less useful for operational settings, like District Chabahar.

### Alert thresholds

C-SUM type alerts using raw counts generally performed better than other alert types. It had the highest AUC, the specific threshold with the highest sum of sensitivity and specificity, and detection delays at least as good as other thresholds tested. Other threshold types also detected all 13 epidemics within two weeks and with at least 90% specificity, but with less sensitivity and lower AUC's. Reducing the minimum duration to three weeks in the gold standard epidemic definition did not alter the relatively superior performance of untransformed C-SUM alerts. Using a five-year instead of a four-year baseline, raw count C-SUM alerts still had higher AUCs than all other alert types except that of raw count Cullen alerts.

No other EDS evaluation has used sensitivity and specificity or ROC areas to compare different alert thresholds. Cullen et al. elected to use means + 2 SDs of monthly counts because of their lower SD but did not otherwise compare this alert with others [6]. Hay et al. [12] also found that untransformed Cullen alerts were more sensitive and less specific than C-SUM or percentile based alerts, but they detected "epidemic years" rather than epidemic periods and the authors questioned whether true epidemics had occurred at all. Later, many of the same authors argued that C-SUM methods may be best because they account for inter-annual variations in the timing of peaks [24]. Teklehaimanot et al. [10] found percentile-based alerts performed as well as the Cullen method, but they did not evaluate the C-SUM method and they required that alerts remain positive for two weeks.

### Limitations

The use of a limited baseline dataset for both generating thresholds and testing them may have contributed to the good performance and the low levels of optimal alert thresholds that we observed, levels which were lower than those used by others [3,4,6,10,12,13]. However, this probably did not cause the observed superior performance of untransformed C-SUM alerts since their relatively superior performance remained robust to changes in the baseline data. The findings need confirmation using a larger baseline dataset to generate epidemic and alert thresholds. The new epidemic definitions and alerts should also be re-evaluated on prospectively collected test data.

The apparent under-representation of females could only have affected results if females' tendency to seek care would periodically increase sufficiently to spuriously inflate reported cases. However, the magnitudes of the observed epidemics make this explanation unlikely.

Wide variations in case counts were observed between centres within Chabahar District. Compared to larger centres, smaller centres mislabelled more spurious aberrations as epidemics when sensitive definitions were used. If less sensitive alert thresholds had been used at centres with fewer indigenous cases, overall specificity may have been improved.

Our methods of defining alerts did not incorporate the use of climate data. Recently, this has been used effectively in predicting epidemics of other vector borne diseases [25]. The malaria epidemic definitions and alert thresholds we used are specific to south-east Iran, which has a very efficient case-finding and reporting system. Still, defining malaria outbreaks based on criteria specific to local circumstances should be possible elsewhere too. This approach to EDS development and evaluation should also be explored in epidemic-prone areas outside Iran.

## Conclusions

In District Chabahar, Iran, malaria epidemics can be defined as a period lasting at least four weeks, when weekly indigenous malaria counts exceed both weekly and overall geometric mean plus one standard deviation. Using this gold standard, the modified raw count C-SUM threshold using mean plus 0.25 SD's identified malaria epidemics within two weeks with the highest sensitivity and specificity. Further study is required to refine the epidemic definition and to confirm which alerts are most sensitive and specific when applied prospectively, both in Iran and in other epidemic prone areas.

## Abbreviations

AUC: Area under the curve; C-SUM: Cumulative sum; EDS: Early detection systems; MCP: Malaria control programmes; MEWS: Malaria early warning systems; PHC: Primary health care; PPC: Potentially preventable cases; ROC: Receiver operating characteristic; SD: Standard deviation.

## Competing interests

The authors declare that they have no competing interests.

## Authors' contributions

WM conducted the background literature search, cleaned the data, conducted the analysis, and drafted the manuscript. AH developed the research question and data analysis strategy, assisted with data analysis, and assisted in the composition and editing of the manuscript. AR helped develop the research question, provided the data and helped to clean it, and helped in editing the manuscript. All authors read and approved the final manuscript.
